# Effect of Prenatal Polycyclic Aromatic Hydrocarbons Exposure on Birth Outcomes: The Polish Mother and Child Cohort Study

**DOI:** 10.1155/2014/408939

**Published:** 2014-07-22

**Authors:** Kinga Polanska, Gerhard Dettbarn, Joanna Jurewicz, Wojciech Sobala, Per Magnus, Albrecht Seidel, Wojciech Hanke

**Affiliations:** ^1^Department of Environmental Epidemiology, Nofer Institute of Occupational Medicine, 8 Teresy Street, 91-348 Lodz, Poland; ^2^Biochemical Institute for Environmental Carcinogens, Prof. Dr. Gernot Grimmer-Foundation, Lurup 4, D-22927 Großhansdorf, Germany; ^3^Division of Epidemiology, Norwegian Institute of Public Health, P.O. Box 4404, Nydalen, N-0403 Oslo, Norway

## Abstract

The aim of this study was to assess the impact of PAH exposure on various anthropometric measures of birth outcomes. The study population consisted of 210 nonsmoking pregnant women. Urine samples collected between 20th and 24th week of pregnancy were used for analysis of the following PAH metabolites: 1-, 2-, 3-, 4-, and 9-hydroxyphenanthrene (1-, 2-, 3-, 4-, and 9-OH-PHE), 1-hydroxypyrene (1-OH-PYR), 1,6 + 1,8-dihydroxypyrene (DI-OH-PYR), phenanthrene *trans*-1,2-dihydrodiol (PHE-1,2-diol), and phenanthrene *trans*-9,10-dihydrodiol (PHE-9,10-diol) by gas chromatography-mass spectrometry. Environmental tobacco smoke exposure (ETS) was assessed by cotinine level in saliva using a stable isotope dilution LC-ESI-MS/MS method. The mean PAH metabolite concentrations were in the range of 0.15 *µ*g/g creatinine for 9-OH-PHE to 5.9 *µ*g/g creatinine for PHE-9,10-diol. It was shown that none of the individual PAH exposure markers demonstrate a statistically significant influence on birth outcomes. Interestingly a statistically significant association was found between the sum of OH-PHE along with cotinine level and the cephalization index after adjusting for potential confounders (*P* = 0.04). This study provides evidence that combined exposure of pregnant women to common environmental pollutants such as PAH and ETS might adversely affect fetal development. Thus, reduction of human exposure to these mixtures of hazardous compounds would in particular result in substantial health benefits for newborns.

## 1. Introduction

Polycyclic aromatic hydrocarbons (PAH) are widespread environmental pollutants that are generated in the course of incomplete combustion and pyrolysis of organic materials such as fuel, coal, wood, garbage, and tobacco. The general population is unavoidably exposed to PAH through polluted ambient air from a variety of sources such as motor vehicles and trucks, coal-fired power plants, residential heating, and environmental tobacco smoke, as well as through contaminated food including charcoal-grilled and smoked meat [1–4].

PAH occur in the environment as a complex mixture of hazardous genotoxic compounds and resulting human exposure raises public health concerns regarding their potential health implications. The prototypic PAH benzo[*a*]pyrene has been classified by the International Agency for Research on Cancer (IARC) as human carcinogen, whereas other PAH are categorized as probably or possibly carcinogenic to humans [1]. Furthermore, reproductive epidemiology studies provide evidence that the developing fetus and infant are sensitive to these environmental toxicants. PAH have been found in placental tissues [5] and umbilical cord blood [6], which suggest that transplacental transfer of these chemicals to the fetus can have a significant impact on fetal development including increased risk of intrauterine growth restriction (IUGR), small-for-gestational age (SGA), and preterm delivery [6–13]. In some studies in addition to anthropometric parameters at birth, such as birth weight and length as well as head and chest circumference, the ponderal index, indicator for thinness of a newborn, and cephalization index, indicator of brain-sparing, were calculated [5, 12, 14]. Both of these indices have been significantly correlated with severe IUGR as well as with poor neurodevelopmental outcomes [15, 16] and subsequent morbidity and mortality [12]. In the study performed by Choi et al. [12] a 1-in-unit increase in prenatal PAH exposure was associated with a 0.04% increase in cephalization index. It has been recently demonstrated that prenatal exposure to PAH may have an effect on the infants' neurodevelopment, including decreased cognitive and motor functions, a reduced child IQ, and an increased risk of behavioral problems [17–21].

The aim of this study was to assess the impact of PAH exposure as determined by urinary biomarkers on various anthropometric measures of birth outcomes based on the Polish Mother and Child Cohort Study (REPRO_PL cohort).

## 2. Material and Methods

### 2.1. Study Design and Population

The present study was based on data from the Polish Mother and Child Cohort (REPRO_PL, http://www.repropl.com/). The mothers' recruitment and follow-up procedures have previously been published in detail [22]. Briefly, subjects were recruited through maternity units or clinics in selected regions of Poland. The recruitment was restricted to women being in single pregnancies, being not assisted with reproductive technology, and having up to 12 weeks of gestation. All women with serious chronic diseases specified in the study protocol were excluded from the study.

The current analysis was focused on pregnant women who were nonsmokers and had no history of occupational PAH exposure. The smoking status of pregnant women was verified by measuring cotinine levels in saliva collected between the 20th and 24th week of gestation using a stable isotope dilution LC-ESI-MS/MS method (high performance liquid chromatography coupled with tandem mass spectrometry/positive electrospray ionization) [23]. All the women with the cotinine levels above 10 ng/mL were considered smokers and were excluded from the analysis. The cut-off point for the cotinine level was selected based on recommendations made by SRNT Subcommittee on Biochemical Verification [24].

The study was approved by the Ethical Committee of the Nofer Institute of Occupational Medicine, Lodz, Poland, and a written consent was obtained from all the subjects.

### 2.2. Exposure Variables

The urine samples were collected from the pregnant women at the time of their second prenatal clinic visit scheduled within the study (between 20th and 24th week of gestation) into polypropylene cups. The samples were stored at −20°C and aliquots were shipped in a frozen state to the Biochemical Institute for Environmental Carcinogens, Prof. Dr. Gernot Grimmer-Foundation, Großhansdorf, Germany, for the analysis of urinary PAH metabolites. The details of analysis of PAH metabolites in biological samples were published elsewhere [25].

1-Hydroxypyrene (1-OH-PYR) was commercially available from Sigma-Aldrich (Taufkirchen, Germany). 1-, 2-, 3-, and 4-hydroxyphenanthrene (OH-PHE), phenanthrene* trans*-1,2-dihydrodiol (PHE-1,2-diol), and phenanthrene* trans*-9,10-dihydrodiol (PHE-9,10-diol) were synthesized as described previously [26]. 9-OH-PHE was purchased from Sigma-Aldrich (Taufkirchen, Germany) and further purified by flash chromatography on silica gel using dichloromethane as an eluent. Both 1,6- and 1,8-dihydroxypyrene were synthesized in accordance with Vollmann et al. (1937) [27]. D_10_-Pyrene and d_12_-chrysene were commercially available from Cambridge Isotope Laboratories Inc. (50 Frontage Road, Andover, USA). 6-Fluoro-1,2-dihydroxy-1,2-dihydrophenanthrene used as an internal standard and all methyl ether derivatives of PAH metabolites necessary for calibration were synthesized at BIU. *β*-Glucuronidase (EC 3.2.1.31)/arylsulfatase (EC 3.1.6.1) from* Helix pomatia* was purchased from Roche Diagnostic GmbH (Mannheim, Germany).

The analyses by gas chromatography-mass spectrometry (GC-MS) were carried out with an Agilent 5890N instrument equipped with a split/splitless injector and an Agilent 5972 mass selective detector (MSD) operated in the single ion monitoring (SIM) mode. For GC separation of the analytes, a Zebron ZB-35 capillary (Phenomenex; 30 m (length) × 0.25 mm (inner diameter), 0.25 *μ*m (film thickness)) was applied.

Determination of phenolic PAH metabolites was performed, as previously described, with several modifications [28]. This methodology has been further developed for a direct determination of PHE-1,2-diol and PHE-9,10-diol. Given the available amount of urine from the Polish Mother and Child Cohort for the present study the number of women for whom the analysis was performed was 210 for PHE-1,2-diol and PHE-9,10-diol and 104 for hydroxyphenanthrenes and hydroxypyrenes.

Creatinine level was measured using the Jaffe colorimetric method with a working range of 0.05–5.00 g creatinine/L. Urine samples with a creatinine concentration lower than 0.3 g/L or higher than 3.0 g/L were excluded from the analysis.

### 2.3. Outcome Variables

The following anthropometric measures at birth, filled in by gynecologists/neonatologists, were considered in the analysis: birth weight (in grams), birth length, and head and chest circumference (in centimeters). In addition, ponderal index (PI) [birth weight (g)/birth length (cm^3^) × 10^2^] and cephalization index (CI) [head circumference (cm)/birth weight (g) × 10^4^] were calculated.

### 2.4. Potential Confounders

The mothers filled in a detailed questionnaire concerning sociodemographic characteristics (age, marital status, parity, maternal education, and employment), medical history, previous and current pregnancies, and lifestyle variables (place of residence, history of passive smoking, alcohol consumption during pregnancy, frequency of grilled or fried food intake based on the food frequency questionnaire, and home characteristics). The questionnaires were administered 3 times during pregnancy (at enrolment: 8th–12th week of gestation, second visit: 20th–24th week of gestation, and third visit: 30th–34th week of gestation) during personal interviews by trained midwives. Factors that could confound the relationship between PAH exposure and anthropometric parameters at birth were selected a priori from a set of characteristics and were tested in a multivariable regression analysis (Table 1). The following potential confounders were considered: gestational age, child gender, passive smoking, and alcohol consumption during pregnancy, maternal age, height, prepregnancy BMI, maternal, marital, educational, and employment status, parity, the place of residence, and season of the last menstrual period. Pregnancy duration was estimated using the date of the last menstrual period (LMP) or ultrasound if it differed from the LMP based estimate by >2 weeks. Environmental tobacco smoke (ETS) exposure was assessed based on the cotinine levels in saliva collected at the same visit as the urine samples for PAH exposure assessment (20th–24th week of gestation) using a stable isotope dilution LC-ESI-MS/MS method [23].

### 2.5. Statistical Analyses

Continuous data were expressed as means ± standard deviations (±SD), as well as the 50th and 95th (for PAH metabolites) percentiles and ranges. Categorical data were presented as numbers and frequencies (%). The creatinine-corrected urinary concentrations of PAH metabolites were analyzed. PAH metabolites and cotinine levels were log-transformed. The 2 following sets of confounders were considered: Model 1: gestational age at birth and child gender; Model 2: all potential confounders significant at a 0.1 level in a multivariable regression (Table 1).

Robust linear regression was performed for each PAH metabolite and for the sum of selected metabolites. In the multiple regression analysis, data were expressed as regression coefficients (*β*) with their standard errors and *P* values. We considered *P* values less than 0.05 (*P* ≤ 0.05) as statistically significant. The statistical analyses were performed using R 3.0.1 statistical package.

## 3. Results 

### 3.1. Characteristics of the Study Participants, Exposure, and Outcomes Variables

Characteristics of mothers, pregnancy, and newborns and their relation to birth outcomes are described in Tables 1 and 2.

The mean maternal age was 28.6 (±3.9) years (Table 1). Most of the women (68%) declared more than 12 years of education and were employed in the first trimester of pregnancy (86%). Also, majority of the mothers were married (82%), and 59% had no children prior to the current pregnancy. The mean prepregnancy BMI was 22.2 kg/m^2^ (±3.6 kg/m^2^). About 70% of the women indicated a big city as their place of residence. About 6% of the women consumed alcohol at least once per month in the pregnancy period. The mean cotinine level in saliva collected in the second trimester of pregnancy was 1.3 ng/mL (±1.3 ng/mL).

About 52% of the children were girls (Table 1). On average the children were born at the 39th week of gestation (±1.4 week) with the mean birth weight of 3417 g (±460 g) and length of 55 cm (±2.8 cm) (Table 2). The mean newborn's head circumference was 34 cm (±1.6 cm) and chest circumference was 33 cm (±1.8 cm).

The mothers who had higher prepregnancy BMI and who were taller had infants with higher birth weight (*P* ≤ 0.02). Boys were bigger and weighed more than girls (*P* ≤ 0.05). Child birth length and head and chest circumference increased along with the increasing maternal height (*P* ≤ 0.03). In addition, an inverse correlation was observed for cotinine level in saliva and child birth head and chest circumference (*P* ≤ 0.06). Gestational age was associated with child birth weight and length (*P* ≤ 0.01).

Characteristics of exposure variables are presented in Table 3. The mean PAH concentrations ranged from 0.15 *μ*g/g creatinine for 9-OH-PHE (±0.2 *μ*g/g creatinine) to 5.9 *μ*g/g creatinine for PHE-9,10-diol (±10.6 *μ*g/g creatinine).

### 3.2. Association between Prenatal PAH Exposure and Various Anthropometric Measures of Birth Outcomes

In the model adjusted for gestational age and newborns' gender, none of the analyzed PAH metabolites was alone associated with any of the anthropometric measures at birth (Table 4). In analysis with adjustment for additional confounders the adverse effect of ∑OH-PYR on chest circumference was of borderline significance (Table 5). Interestingly, a significant interaction effect of the sum of OH-PHE and the cotinine level on cephalization index was noticed after adjusting for gestational age, newborns' gender, maternal prepregnancy BMI, and maternal height (Table 5), indicating that combined high PAH and high ETS exposure positively correlated with the ratio of head circumference to the birth weight.

The same pattern was observed for the sum of PHE-diol, but the association was of borderline significance (*P* for interaction = 0.06).

## 4. Discussion

In this study we have not found any statistically significant effects of individual urinary biomarker of PAH exposure on birth outcomes. However, a significant interaction effect of the sum of OH-PHE and the cotinine level in saliva as a measure of PAH and ETS exposure, respectively, on the cephalization index was noticed (*P* = 0.04). This association is of potential concern since poor pregnancy outcomes may be linked to later neurodevelopmental and other health problems.

In our previous analysis (with 20% of the population classified as smokers) the association between 1-OH-PYR and biometric parameters at birth was not statistically significant after controlling for the cotinine level in saliva as the biomarker of smoking status [14]. Taking into consideration the smoking status as significant confounder, the current analysis was restricted to the nonsmoking women with cotinine levels in saliva below 10 ng/mL [24]. Another very important feature of the current study, compared to the previous one, is the analysis of a variety of different urinary PAH metabolites as biomarkers in addition to 1-OH-PYR, which has resulted in a more comprehensive assessment of PAH exposure.

### 4.1. Exposure Assessment

A variety of different PAH metabolites in the urine collected during the second trimester of pregnancy were selected as the biomarkers of exposure. There have been concerns whether the single spot urine tests represent the long-term exposure. The analysis performed by Choi et al. (2008) indicated that measurements during the third trimester were significantly lower compared with the second trimester, whereas those during the first trimester were not [29]. However, our previous analysis based on a subsample of the REPRO_PL cohort indicated no differences between 1-OH-PYR levels in urine collected during the second and third trimesters of pregnancy (second trimester 1-OH-PYR geometric mean (GM) = 0.4 ± 2.4 *μ*g/g creatinine and third trimester GM = 0.4 ± 2.4 *μ*g/g creatinine; *P* = 0.7) [30].

Although the studies evaluating the impact of PAH exposure on pregnancy outcomes exist, it is difficult to compare their results to these obtained in the current study. This results from the fact that researchers select different PAH metabolites for their analysis so direct comparison of the magnitude of exposure is not possible. However, based on the analysis by Choi et al. (2006), personal airborne PAH exposure was 10-fold higher in Krakow than in NYC [11]. In addition, multiple linear regression analysis performed by Tonne at al. (2004) on pregnant minority women in NYC revealed associations between personal PAH exposures and season of the year, time spent outdoors, residential heating, and indoor burning of incense [31]. Our current results (although based on different biomarkers of exposure) also indicate high PAH exposure. The median 1-OH-PYR level observed in the pregnant women's urine (0.35 *μ*g/g creatinine) was higher than that noted in the INMA cohort of nonsmoking pregnant women in Valencia (0.1 *μ*g/g creatinine) and postpartum nonsmoking women from Saudi Arabia (0.05 *μ*g/g creatinine) [5, 32]. The Human Biomonitoring Commission of the German Federal Environment Agency established 1-OH-PYR levels in urine of 0.3 *μ*g/g creatinine as the reference value for the nonsmoking general population aged between 3 and 69 years [33, 34]. In the present study, this reference value was much higher (1.0 *μ*g/g creatinine). It is important to notice that most of the women included in our analysis lived in a big city (Lodz) which can be the reason for the higher 1-OH-PYR level. This was confirmed by the stationary ambient air monitoring data (from Voivodeship Inspectorates for Environmental Protection), which indicated that the annual mean BaP level in various locations within Lodz was substantially higher than the target value of 1 ng/m^3^ recently set into force by the European Commission [35, 36].

### 4.2. Effect of Exposure on Neonatal Anthropometric Measures

The association between urinary PAH metabolite levels or PAH-DNA damage and reduced biometric measures at birth has been reported in some, but not all, studies. For example, the study performed in the Czech Republic indicated that ambient PAH exposure in the early stage of pregnancy significantly increased the risk of intrauterine growth restriction (OR = 1.2; 95% CI 1.1–1.4) [7]. The analysis performed in Poland (Krakow and Limanowa) by Perera et al. (1998), with high PAH exposure levels, indicated that newborns whose levels of PAH-DNA adducts were above the median had significantly decreased birth length and weight and head circumference [8]. Determination of airborne PAH by personal air sampling indicated that such exposure was associated with significantly reduced birth weight in Krakow Caucasians (*P* < 0.01) and in NYC African Americans (*P* < 0.05), but not in NYC Dominicans [9, 11]. A recently published study indicated that PAH exposure appears to exert the greatest adverse effect on fetal growth during the first trimester [13]. This kind of analysis was not feasible in our study. No association was found between 1-OH-PYR levels and any of pregnancy outcomes in the two recent studies conducted in Saudi Arabia and Japan [5, 37].

The impact of PAH exposure on the cephalization index was reported in an earlier study by Choi et al. (2008) [12]. Increased cephalization index, the ratio of head size to birth weight, may suggest that growth restriction might induce a “brain-sparing” process at developmental expense of other organs. This could be of particular concern taking into account that children born with a larger head circumference relative to the body weight score significantly lower in neurodevelopmental tests, IQ, and school performance and are at a greater risk of subsequent morbidity and mortality [12]. Perera et al. (2004) reported that combined exposure to high ETS and high BaP-DNA adducts had a significant multiplicative effect on birth weight (*P* = 0.04) and head circumference (*P* = 0.01) [38]. In the present study the interaction effect of PAH metabolites and cotinine was significant only for the cephalization index; for other birth outcomes, the pattern of association, although not statistically significant, was similar to that observed by Perera et al. (2004) [38].

Fetal toxicity caused by PAH and ETS may result from antiestrogenic effects [39], binding of constituents to human aryl hydrocarbon receptor and subsequently inducing cytochrome P450 enzymes [40], DNA damage [41–43], and binding of receptors for placental growth factors resulting in a decreased exchange of oxygen and nutrients or direct effect of carbon monoxide [7, 38].

### 4.3. Confounders

Majority of the studies published in this field have focused on the known possible confounding variables such as child gender, gestational age, marital and educational status of pregnant women, prepregnancy BMI, and season of the last menstrual period, which were also addressed in our analysis. By restricting our study population to healthy women without serious pregnancy complications and to single pregnancies, we were able to eliminate several confounding factors.

### 4.4. Strengths and Limitations

The current analysis is based on the Polish Mother and Child Cohort Study (REPRO_PL cohort). The prospective study design with well-assessed exposure levels based on biomarker measurements (cotinine, PAH metabolites) is an important advantage of the current analysis. Thanks to it we have addressed some of the traditional limitations such as exposure misclassification and retrospective or cross-sectional exposure assessment. Additionally, a series of detailed questionnaires (collected once in each trimester of pregnancy and one week after delivery) allow for reliable assessment of coexposure and confounding variables.

The current study results may be limited by a modest sample size; however it has sufficient power to perform the presented analysis. As it was mentioned above, the biomarkers were measured only at a single time point which may constitute an additional limitation of our study. Finally, although creatinine correction is commonly used for urinary biomarkers, in some studies the specific gravity was used instead of creatinine in order to adjust PAH metabolites for urine dilution. However, specific gravity is highly correlated with creatinine [44]. Therefore an overcorrection for urine dilution is not expected in this study, especially because the results from the very diluted urine samples have been discarded.

## 5. Conclusions 

This study provides evidence that combined exposure of pregnant women to common environmental pollutants such as PAH and ETS might adversely affect fetal development. Thus, reduction of human exposure to these mixtures of hazardous compounds would in particular result in substantial health benefits for newborns and improve prevention of restrictions in their subsequent development.

## Figures and Tables

**Table 1:** Characteristics of the pregnant women, pregnancies, and the newborns and their associations with birth outcomes (multivariable regression analysis).

Characteristic^a^	Mean or *n*	±SD or %	β (SE)					
			*P*					
			Birth weight	Birth length	Birth head circumference	Birth chest circumference	Ponderal index	Cephalization index
Gestational age (weeks); mean, SD (*N* = 210)	39.2	1.4	111.9 (41.3) **0.008**	0.7 (0.3) **0.01**	0.08 (0.1) 0.6	0.3 (0.2) **0.08**	0.01 (0.02) 0.7	−3.1 (1.0) **0.003**
Maternal age (years); mean, SD (*N* = 210)	28.6	3.9	4.3 (14.1) 0.8	0.2 (0.1) **0.09**	0.05 (0.05) 0.3	0.03 (0.06) 0.6	−0.01 (0.01) **0.08**	0.04 (0.3) 0.9
Prepregnancy BMI (kg/m^2^); mean, SD (*N* = 210)	22.2	3.6	27.5 (11.7) **0.02**	0.005 (0.08) 0.9	0.07 (0.04) **0.08**	0.08 (0.05) **0.07**	0.01 (0.006) **0.07**	−0.6 (0.3) **0.03**
Maternal height (cm); mean, SD (*N* = 210)	167	6.0	21.9 (7.5) **0.004**	0.2 (0.05) **0.003**	0.08 (0.03) **0.003**	0.07 (0.03) **0.03**	−0.004 (0.004) 0.3	−0.4 (0.2) **0.01**
Cotinine (ng/mL); mean, SD (*N* = 210)	1.3	1.3	−30.6 (48.2) 0.5	−0.3 (0.3) 0.4	−0.4 (0.2) **0.02**	−0.4 (0.2) **0.06**	0.01 (0.03) 0.3	−0.2 (1.1) 0.8
Marital status (*N* = 210)								
Single	37	17.6	−27.8 (123.1)	0.4 (0.8)	0.3 (0.4)	0.03 (0.4)	−0.03 (0.07)	1.5 (2.9)
Married	173	82.4	0.8	0.7	0.5	0.9	0.7	0.6
Maternal education (years of education) (*N* = 209)								
>12	141	67.5	153.8 (111.9)	−0.3 (0.7)	0.4 (0.4)	0.02 (0.4)	0.1 (0.06)	−4.4 (2.7)
≤12	68	32.5	0.2	0.7	0.3	0.9	**0.08**	0.2
Employment status in the first trimester (*N* = 209)								
Employed	179	85.6	−55.0 (135.5)	−0.7 (0.9)	−0.2 (0.5)	−0.5 (0.5)	0.04 (0.07)	1.1 (3.2)
Unemployed	30	14.4	0.7	0.4	0.8	0.3	0.6	0.7
Parity (*N* = 210)								
≥1	87	41.4	148.8 (116.3)	0.4 (0.8)	0.3 (0.4)	1.2 (0.5)	0.03 (0.06)	−3.2 (2.8)
0	123	58.6	0.2	0.6	0.5	**0.003**	0.6	0.3
Season of the last menstrual period (*N* = 210)								
October–April	110	52.4	−5.0 (90.0)	0.1 (0.6)	−1.1 (0.3)	−0.4 (0.3)	−0.001 (0.05)	−2.8 (2.1)
May–September	100	47.6	0.9	0.8	**0.001**	0.3	0.9	0.2
Place of residence (*N* = 210)								
>100 thousand inhabitants	146	69.5	7.7 (99.0)	0.4 (0.7)	−0.4 (0.4)	0.5 (0.4)	−0.03 (0.05)	−0.9 (2.4)
≤100 thousand inhabitants	64	30.5	0.9	0.5	0.3	0.3	0.6	0.7
Alcohol consumption during pregnancy (frequency) (*N* = 206)								
At least once per month	13	6.2	−63.0 (170.2)	0.4 (1.1)	−0.1 (0.6)	−0.9 (0.7)	−0.1 (0.1)	0.6 (4.1)
Less than once per month	193	93.8	0.7	0.7	0.8	0.2	0.4	0.9
Newborns' gender (*N* = 210)								
Female	109	51.9	−176.0 (88.6)	−1.9 (0.6)	−0.9 (0.3)	−0.7 (0.3)	0.08 (0.05)	3.0 (2.1)
Male	101	48.1	**0.05**	**0.002**	**0.006**	**0.05**	**0.09**	0.2

Numbers may not sum up 210 in all the variables because some values are missing.

Mean and standard deviation (±SD) are presented for continuous variables. *N* and % are presented for categorical variables.

^a^For categorical variables the second category is the reference category in the regression analysis.

*β*: beta coefficient (and SE standard error) from the multivariable regression analysis.

*P* : *P* value from Wald in the multivariable regression analysis.

**Table 2:** Newborn anthropometric indicators.

	Median	Mean	±SD	Range
Birth weight (g) *N* = 210	3400.0	3417.3	460.3	2150.0–5100.0
Child length (cm) *N* = 210	55.0	55.2	2.8	48.0–64.0
Head circumference (cm) *N* = 208	34.0	34.3	1.6	31.0–39.0
Chest circumference (cm) *N* = 208	33.0	33.3	1.8	27.0–39.0
Ponderal index (g/cm^3^) *N* = 207	2.0	2.0	0.2	1.5–2.8
Cephalization index (cm/g) *N* = 208	100.5	101.9	12.2	74.5–148.8

**Table 3:** Descriptive statistics of the PAH metabolites (*μ*g/g creatinine).

PAH metabolites	*N*	Mean	±SD	Median	95th percentile	Range
1-OH-PHE	104	1.50	1.27	1.10	4.14	0.30–8.64
2-OH-PHE	104	0.61	0.50	0.43	1.47	0.13–3.07
3-OH-PHE	104	0.45	0.43	0.31	1.27	0.05–2.79
4-OH-PHE	104	0.17	0.14	0.13	0.48	0.02–0.70
9-OH-PHE	104	0.15	0.20	0.10	0.43	0.004–1.70
∑OH-PHE	104	2.88	2.43	2.12	7.26	0.61–16.77
PHE-1,2-diol	210	4.09	4.53	2.41	12.70	0.06–26.72
PHE-9,10-diol	210	5.93	10.57	2.70	23.88	0.05–94.15
∑PHE-diol	210	10.01	13.35	6.05	34.44	0.24–115.35
1-OH-PYR	104	0.43	0.30	0.35	0.97	0.06–1.64
DI-OH-PYR	104	0.30	0.33	0.21	0.84	0.002–2.52
∑OH-PYR	104	0.73	0.57	0.56	1.75	0.06–4.17

1-, 2-, 3-, 4-, and 9-OH-PHE, 1-, 2-, 3-, 4-, and 9-hydroxyphenanthrene; ∑OH-PHE, sum of 1-, 2-, 3-, 4-, and 9-OH-PHE; PHE-1,2-diol, phenanthrene *trans*-1,2-dihydrodiol; PHE-9,10-diol, phenanthrene *trans*-9,10-dihydrodiol; ∑PHE-diol, sum of PHE-1,2-diol and PHE-9,10-diol; 1-OH-PYR, 1-hydroxypyrene; DI-OH-PYR, sum of 1,6- and 1,8-dihydroxypyrenes; ∑OH-PYR, sum of 1-OH-PYR and DI-OH-PYR.

**Table 4:** Association between PAH metabolites and anthropometric measures at birth (adjusted for gestational age and the newborns' gender).

PAH metabolites∗	Birth weight (g)			Cephalization index (cm/g)			Chest circumference (cm)			Child length (cm)			Head circumference (cm)			Ponderal index (g/cm^3^)		
	*β*	SE	P	β	SE	P	β	SE	P	β	SE	P	β	SE	P	β	SE	P
1-OH-PHE	−76	66	0.2	1.5	1.6	0.3	−0.4	0.3	0.2	0.1	0.4	0.8	−0.2	0.3	0.5	−0.05	0.03	0.1
2-OH-PHE	−59	66	0.4	1.1	1.6	0.5	−0.3	0.3	0.3	0.04	0.4	0.9	−0.2	0.3	0.4	−0.03	0.03	0.4
3-OH-PHE	−46	53	0.4	1.4	1.3	0.3	−0.04	0.2	0.9	0.1	0.4	0.8	−0.01	0.2	1	−0.03	0.03	0.3
4-OH-PHE	−62	57	0.3	1.9	1.4	0.2	−0.2	0.2	0.4	−0.2	0.4	0.5	−0.1	0.2	0.6	−0.02	0.03	0.5
9-OH-PHE	−43	47	0.4	1.5	1.1	0.2	−0.1	0.2	0.6	0.1	0.3	0.8	0.1	0.2	0.7	−0.02	0.02	0.4
∑OH-PHE	−69	66	0.3	1.5	1.6	0.3	−0.3	0.3	0.3	0.1	0.4	0.8	−0.1	0.3	0.6	−0.04	0.03	0.2
PHE-1,2-diol	7	27	0.8	−0.2	0.7	0.8	−0.1	0.1	0.3	0.1	0.2	0.7	−0.03	0.1	0.8	−0.00	0.01	0.8
PHE-9,10-diol	−19	23	0.4	0.2	0.6	0.7	−0.1	0.1	0.5	−0.01	0.2	1	−0.1	0.1	0.2	−0.01	0.01	0.4
∑PHE-diol	−14	29	0.6	0.2	0.7	0.8	−0.1	0.1	0.3	0.03	0.2	0.9	−0.1	0.1	0.3	−0.02	0.02	0.3
1-OH-PYR	−26	62	0.7	0.01	1.5	1	−0.3	0.3	0.2	−0.2	0.4	0.6	−0.3	0.2	0.3	−0.01	0.03	0.7
DI-OH-PYR	−13	38	0.7	0.03	0.9	1	−0.1	0.2	0.4	0.1	0.3	0.8	−0.1	0.1	0.5	−0.02	0.02	0.3
∑OH-PYR	−31	58	0.6	0.2	1.4	0.9	−0.3	0.2	0.2	−0.1	0.4	0.8	−0.2	0.2	0.3	−0.02	0.03	0.5

*β*: beta coefficient (and SE standard error).

*P*:*P* value.

*PAH metabolites were log-transformed.

**Table 5:** Association between PAH metabolites and anthropometric measures at birth by multiple linear regression.

PAH metabolites∗	Birth weight (g)				Cephalization index (cm/g)				Chest circumference (cm)				Child length (cm)				Head circumference (cm)				Ponderal index (g/cm^3^)			
	*β*	SE	*P*	PAH and ETS interaction *P*	*β*	SE	*P*	PAH and ETS interaction *P*	*β*	SE	*P*	PAH and ETS interaction *P*	*β*	SE	*P*	PAH and ETS interaction *P*	*β*	SE	*P*	PAH and ETS interaction *P*	*β*	SE	*P*	PAH and ETS interaction *P*
∑OH-PHE	−45	65	0.5	0.2	0.7	1.6	0.6	**0.04**	−0.4	0.3	0.2	0.5	0.1	0.4	0.8	0.3	−0.1	0.2	0.6	0.2	−0.03	0.03	0.4	0.8
∑PHE-diol	−6	29	0.8	0.1	0.2	0.8	0.8	0.06	−0.2	0.1	0.2	0.3	−0.03	0.2	0.9	0.08	−0.2	0.1	0.2	0.9	−0.01	0.02	0.6	0.2
∑OH-PYR	−15	56	0.8	0.3	−0.3	1.3	0.8	0.2	−0.4	0.2	0.06	0.2	−0.1	0.4	0.9	0.5	−0.1	0.2	0.5	0.7	−0.01	0.03	0.7	0.6

Adjusted for: Birth weight: gestational age, newborns' gender, maternal pre-pregnancy BMI, maternal height.

Birth length: gestational age, newborns' gender, maternal age, maternal height.

Birth head circumference: gestational age, newborns' gender, maternal pre-pregnancy BMI, maternal height, log transformed cotinine level during pregnancy (except PAH and ETS interaction analysis), season of last menstrual period.

Birth chest circumference: gestational age, newborns' gender, maternal pre-pregnancy BMI, maternal height, log transformed cotinine level during pregnancy (except PAH and ETS interaction analysis), parity.

Ponderal index: gestational age, newborns' gender, maternal age, maternal pre-pregnancy BMI, maternal education.

Cephalization index: gestational age, newborns' gender, maternal pre-pregnancy BMI, maternal height.

*β*-beta coefficient (and SE standard error).

*P*-*P*-value.

*PAH metabolites were log transformed.
